# The effects of altitude/hypoxic training on oxygen delivery capacity of the blood and aerobic exercise capacity in elite athletes – a meta-analysis

**DOI:** 10.20463/jenb.2016.03.20.1.3

**Published:** 2016-03-31

**Authors:** Hun-young Park, Hyejung Hwang, Jonghoon Park, Seongno Lee, Kiwon Lim

**Affiliations:** 1 Physical Activity and Performance Institute (PAPI), Konkuk University, SeoulRepublic of Korea; 2 Department of Physical Education, Korea University, SeoulRepublic of Korea; 3 Department of Physical Education, Hanyang University, SeoulRepublic of Korea; 4 Department of Physical Education, Konkuk University, SeoulRepublic of Korea

**Keywords:** altitude/hypoxic training, oxygen delivery capacity of the blood, aerobic exercise capacity, meta-analysis, heterogeneity, fixed and random effect model

## Abstract

**[Purpose]:**

This study was designed as a meta-analysis of randomized controlled trials comparing effectiveness of altitude/hypoxic training (experimental) versus sea-level training (control) on oxygen delivery capacity of the blood and aerobic exercise capacity of elite athletes in Korea.

**[Methods]:**

Databases (Research Information Service System, Korean studies Information Service System, National Assembly Library) were for randomized controlled trials comparing altitude/hypoxic training versus sea-level training in elite athletes. Studies published in Korea up to December 2015 were eligible for inclusion. Oxygen delivery capacity of the blood was quantified by red blood cell (RBC), hemoglobin (Hb), hematocrit (Hct), erythropoietin (EPO); and aerobic exercise capacity was quantified by maximal oxygen consumption (VO_2_max). RBC, Hb, Hct, VO_2_max represented heterogeneity and compared post-intervention between altitude/hypoxic training and sea-level training in elite athletes by a random effect model meta-analysis. EPO represented homogeneity and meta-analysis performed by a fixed effect model. Eight independent studies with 156 elite athletes (experimental: n = 82, control: n = 74) were included in the metaanalysis.

**[Results]:**

RBC (4.499×10^5^ cell/ul, 95 % CI: 2.469 to 6.529), Hb (5.447 g/dl, 95 % CI: 3.028 to 7.866), Hct (3.639 %, 95 % CI: 1.687 to 5.591), EPO (0.711 mU/mL, 95% CI: 0.282 to 1.140), VO_2_max (1.637 ml/kg/min, 95% CI: 0.599 to 1.400) showed significantly greater increase following altitude/hypoxic training, as compared with sea-level training.

**[Conclusion]:**

For elite athletes in Korea, altitude/ hypoxic training appears more effective than sea-level training for improvement of oxygen delivery capacity of the blood and aerobic exercise capacity.

## INTRODUCTION

Since the 1968 Mexico City Olympics, studies have been conducted to perform training in altitude/hypoxic environment for the enhanced performance of athletes; currently, these training methods are commonly being applied to many athletes and coaches^45^. In the past, training in altitude/hypoxic environment was only performed at high altitude areas, including Chamonix in France, Albuquerque in United States, and Kunming in China; however, a variety of artificial altitude/hypoxic environment (normobaric hypoxia, hypobaric hypoxia) equipment, such as hypoxic masks, hypoxic tents, hypoxic trucks, hypoxic hotels, and hypoxic training centers have been developed since the 1990s^4, 17, 25, 26, 29^.

The performance at sea-level can be improved by 3 ways through the training in both natural and artificial altitude/hypoxic environments45. Living High Training High (LHTH), was the first design of living and training at 1500 - 4000 m in the natural altitude environments that enhances Red Blood Cell (RBC) count, Hemoglobin (Hb) concentration, Hematocrit (Hct), Maximal Oxygen Consumption (VO_2_max), and exercise performance at sea-level. In particular, LHTH is constantly used for the enhanced performance in countries with natural altitude environments, including Kenya and Ethiopia. In addition, many elite-athletes in other countries plan to participate in the training camps in altitude environments such as Albuquerque in United States, Kunming in China, and Chamonix in France. However, LHTH has a major limitation, which is failure to perform training of the same intensities (e.g., running speed), as compared with sea-level training. Buskirk et al.^6^ reported that the collegiate distance runners who completed 880 yard, 1 mile, and 2 mile runs in the LHTH decreased by 3 - 8 % in exercise performance. Moreover, several studies demonstrated that absolute training intensity during continuous and interval workout was significantly decreased at 2500 m, as compared with sea-level^19, 24^.

The living high training low (LHTL) was developed by Dr. Benjamin Levine and James Stray-Gundersen of the United States in the early 1990s, as a potential modification to the limitation of LHTH. Basically, LHTL simultaneously offers athletes the beneficial effects of altitude/ hypoxic acclimation (e.g., increased RBC count and Hb concentration) and sea-level training (i.e., maintenance of training intensity). In addition, LHTL has shown the efficacy of enhancing athletic performances and records, resulting in positive hematological, metabolic, and neuromuscular adaptations^5, 18, 26, 28^. Elite athletes use living high at 2000 - 3000 m and simultaneously training low below 1500 m^37, 43, 44^. LHTL is performed not only in natural altitude environments but also a variety of artificial hypoxic environments. Particularly, these artificial hypoxic environments can be accomplished using a several methods and devices (e.g., nitrogen dilution, oxygen filtration, and supplemental oxygen)^1, 5, 10, 12, 23, 28, 29, 32, 34, 46^.

Last, in living low training high (LLTH), athletes live at sea-level and are exposed to relatively short intervals (< 180 min) of intermittent hypoxic exposure (IHE) through the resting state and intermittent hypoxic training (IHT) during workout. LLTH reportedly enhances exercise performance by stimulating an increase in serum erythropoietin (EPO), RBC count, skeletal muscle mitochondrial density, capillary-to-fiber ratio, fiber cross-sectional area via upregulation of hypoxia-inducible factor 1α (HIF-1α)^9, 16, 27, 33, 42^. Taken together, the empirical evidence regarding the efficacy and physiological changes of LLTH is used for athletic performance and record in the sea-level environments in several countries. Particularly, it plays an important role in increased athletic performance in altitude/ hypoxic environments.

A number of studies on training have shown enhanced aerobic exercise capacity and athletic performance in natural altitude/artificial hypoxic environments. The purpose of these studies are to elucidate the effect of training and provide the proper types of altitude/hypoxic training through the frequency of exposure, training altitude, characteristics of subjects, and types of training in the systematic review and meta-analysis^3, 4, 17, 30, 41^. However, it is still controversial whether these studies in other countries are applicable to Korean elite-athlete due to racial and physiological differences. Furthermore, in Korea, the experimental studies have reported on elite-level athlete in natural altitude and artificial hypoxic environments trainings in 2000s, however, evidence of the training effectiveness based on systematic review and meta-analysis is currently not available.

The purpose of this study was to determine the comprehensive efficacy of oxygen delivery capacity of the blood and aerobic exercise capacity in natural altitude and artificial hypoxic environments training for enhanced athletic performance by meta-analysis in Korea. Meta-analysis is a set of statistical methods for combining quantitative results from multiple studies to produce an overall summary of empirical knowledge on a given topic. It is used to analyze central trends and variations in results across studies, and minimize error and bias in the study^20^. In other words, although the previous results of studies show conflict of interests or positive effects, the meta-analysis elucidates the effect of direction and size depending on effect size20. Therefore, it may propose empirical training methods, altitude/ hypoxic environment system, and enhanced athletic performance for athlete, coach, and researcher.

## METHODS

### Study design

This study determined effectiveness of natural altitude and artificial hypoxic training based on the results of oxygen delivery capacity of the blood (e.g., RBC, Hb, Hct, and EPO) and aerobic exercise capacity (e.g., VO_2_max) in Korean athletes through meta-analysis.

### Reference search and data extraction

All relevant studies in systematic reviews and meta-analyses via PICOS (Participants, Interventions, Comparisons, Outcomes, and Study Design) on the Cochrane guidelines were selected^13^. Furthermore, for the systematic review and aggregate data meta-analysis using by PRISMA flowchart with 5 phases, we considered eligible studies that investigated the effects on oxygen delivery capacity of the blood and aerobic exercise capacity in altitude/hypoxic environments.

**Figure 1.**
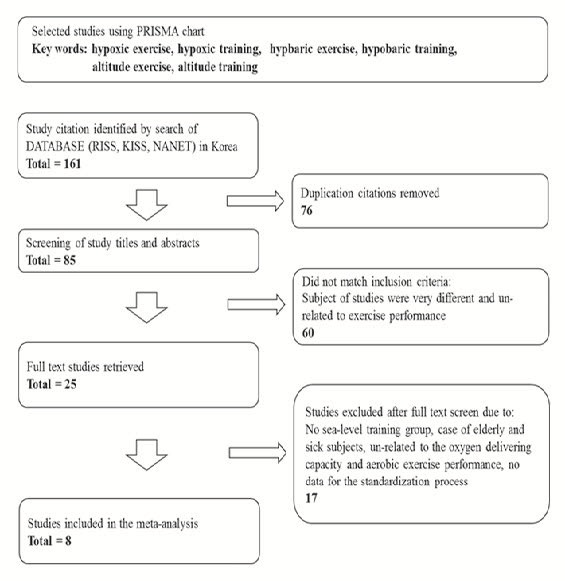
PRISMA chart of the search and study inclusion process

We identified relevant studies through a database of Research Information Service System (RISS), Korean studies Information Service System (KISS), and National Assembly Library (NANET) without any publication year restriction until December 15, 2015. We further identified studies for confidence by reviewing the reference lists of KCI in the field to identify published data only (Figure 1). We used 161 citations to collect information on the following: hypoxic exercise, hypoxic training, hypobaric exercise, hypobaric training, altitude exercise, and altitude training. From a total of 161 eligible studies identified, 85 were included in the aggregate data meta-analysis and 76 were excluded due to the overlapping study designs after title/abstract scan. In addition, 60 that were not relevant determinants or outcome data for enhanced athletic performance were excluded. Thus, 25 retrieved selected full texts were reviewed so that the excluded were as follows: not elite athletes (e.g. healthy humans, the elderly, and patients), no control group, not relevant dependent variables on oxygen delivery capacity of the blood and aerobic exercise capacity, and no data used in the meta-analyses (e.g., mean, standard deviation, and sample size). Therefore, based on study characteristics of 161 references initially identified, 8 were included in the aggregate meta-analysis.

### Characteristics and variables of selected references

The 8 selected references were classified according to authors, published year, characteristics of subjects, number of subjects, and altitude/hypoxic environments training (e.g., type, duration, and frequency) (Table 1) and the number of subjects were 156 subjects (exercise group: 82 and control group: 74). All studies were conducted on elite athletes: 1 of high school soccer players, 2 of high school track players, 1 of national level fin swimmers, 1 of collegiate tennis players, 1 of national level swimmers, 1 of collegiate track players, and 1 of collegiate basketball players. Additionally, types of altitude/hypoxic environments training consisted of 2 LHTH, 1 LHTL, and 5 LLTH.

Of these 8 studies, meta-analyses included 8 oxygen delivery capacity of the blood (e.g., RBC, Hb, and Hct), 5 EPO, and 7 aerobic exercise capacity, in order to determine the comprehensive efficacy of oxygen delivery capacity of the blood and aerobic exercise capacity in altitude/hypoxic environments training for the enhanced athletic performance.

**Table 1.** Characteristics of included studies for meta-analysis

Study	N	Sex	Subject	Altitude/Hypoxic training type
Sunoo et al. (2007)	Exp. (11) Con. (10)	Male and female	Fin swimmers	- LLTH(IHT): 3,000m simulated altitude - 80%HRmax exercise intensity - 30min treadmill exercise and 30min bike exercise - 1hrs, 4days/week, 3weeks
Shin & Cho (2003)	Exp. (8) Con. (8)	Male	Swimmers	- LLTH(IHT): 2,500m simulated altitude - aerobic exercise - 1hrs, 5days/week, 4weeks
Yun & Lee (2014)	Exp. (8) Con. (8)	Male	Tennis players	- LLTH(IHT): 2,500m~4,000m simulated altitude increased by 500m in every week - resting, aerobic and anaerobic exercise - 6hrs, 3days/week, 4weeks
Park et al. (2011)	Exp. (10) Con. (10)	Male	Athletes	- LHTL: Living(3,000m), Training(700~1,330m) more than16 hrs residence everyday(living) - aerobic and interval exercise(training), 4hrs, 6day/week, 4weeks
Sunoo & Hwang (2004)	Exp. (8) Con. (7)	Female Female (5) Male (2)	Athletes	- LHTH: 3,000m simulated altitude living(8hrs), 2,000m simulated altitude training(1.5hrs) - 70%VO_2_max training, 4days/week, 6weeks
Sunoo et al. (2005)	Exp. (9) Con. (9)	males	Basketball players	- LLTH(IHE): 3,000m simulated altitude resting and sleeping - 8hrs, 7days/week, 3weeks
Jung et al. (2004)	Exp. (10) Con. (10)	males	Athletes	- LHTH: living and training in 1,896m altitude 60~70%HRmax exercise, 7.5hrs, 6days/week, 4weeks - resting and sleeping at the remaining non-exercise time
Kim et al. (2009)	Exp. (18)	males	Soccer players	- LLTH(IHE) : 3,000m simulated altitude resting and sleeping - 8hrs, 7days/week, 4weeks

### Statistical analysis

All statistical analyses were performed with Excel (Microsoft, USA) and CMA version 3.0 (Biostat, USA).

We used Cohen’s d where the term effect size can refer to the value of a statistic calculated from a sample of data and standardized mean differences^8^. However, a lower Cohen’s d indicates the necessity of Hedges’g due to a bias of the overestimated effect size, vice versa, as can subsequently be converted to g with the larger sample size and lower sample size^20^. This meta-analysis calculated the effect size of studies that converted from Cohen’s d to Hedges’g with correction factor.

A Q-statistic and Higgins’ I2 statistic were employed to provide a test of statistical homogeneity for the differences in effect sizes among studies. Under the fixed-effect model we calculated the weighted effect size (weighted mean difference: WMD) if the test of homogeneity was statistically significant, and vice versa, we allowed the random effect model if the test of heterogeneity was significant.

A significance level of a < 0.05 was used to determine statistical difference for mean of effect size and the confidence interval was reflected at a confidence level of 95 %.

## RESULTS

### The effect of altitude/hypoxic training on oxygen delivery capacity of the blood

Eight studies were selected for the effect of altitude/hypoxic training on RBC, Hb, Hct and 5 studies for the effect of altitude/hypoxic training on EPO. Among oxygen delivery capacity of the blood, heterogeneity was identified in RBC (Q-value = 106.578, p = .000, I2 = 93.432) and effect size calculated by random effect model. Elite athletes in the altitude/hypoxic training group improved their RBC by 4.499×105 cell/μl (95 % CI: 2.469 - 6.529, p = .000) more than the sea-level training group (Table 2). Heterogeneity was also identified in Hb (Q-value = 119.043, p = .000, I2 = 94.120) and effect size calculated by random effect model. Elite athletes in the altitude/hypoxic training group improved their Hb by 5.447 g/dl (95% CI: 3.028 - 7.866, p = .000) more than the sea-level training group (Table 3). Likewise, heterogeneity was identified in Hct, (Q-value = 104.973, p = .000, I2 = 93.332) and effect size calculated by random effect model. Elite athletes in the altitude/hypoxic training group improved their Hct by 3.639 % (95% CI: 1.687 - 5.591, p = .000) more than the sea-level training group (Table 4). However, in EPO, homogeneity was identified (Q-value = 2.115, p = .715, I2 = .000) and effect size calculated by fixed effect model. Elite athletes in the altitude/hypoxic training group improved their EPO by 0.711 mU/mL (95% CI: 0.282 - 1.140, p = .001) more than the sea-level training group (Table 5).

**Table 2.** Effects of altitude/hypoxic training vs. sea-level training on RBC (10^5^cell/μl).

Model	Study name	Std diff in means	standard error	variance	Lower limit	Upper limit	Z-value	p-value
	Sunoo et al. (2007)	1.453	0.491	0.241	0.490	2.415	2.958	0.003
	Shin and Cho (2003)	31.752	5.635	31.756	20.707	42.797	5.635	0.000
	Yun and Lee (2014)	2.832	0.708	0.501	1.445	4.219	4.003	0.000
	Park et al. (2011)	7.062	1.203	1.447	4.705	9.420	5.871	0.000
	Sunoo and Hwang (2004)	0.761	0.536	0.287	-0.289	1.811	1.420	0.156
	Sunoo et al. (2005)	2.292	0.607	0.368	1.103	3.482	3.778	0.000
	Jung et al. (2004)	0.632	0.458	0.210	-0.266	1.531	1.380	0.168
	Kim et al. (2009)	15.891	2.085	4.348	11.804	19.978	7.621	0.000
Random		4.499	1.036	1.073	2.469	6.529	4.343	0.000

Heterogeneity: Q-value=106.572(p=.000), I^2^=93.432

**Table 3.** Effects of altitude/hypoxic training vs. sea-level training on Hb (g/dl).

Model	Study name	Std diff in means	standard error	variance	Lower limit	Upper limit	Z-value	p-value
	Sunoo et al. (2007)	1.453	0.491	0.241	0.490	2.415	2.958	0.003
	Shin and Cho (2003)	96.449	17.057	290.953	63.018	129.881	5.654	0.000
	Yun and Lee (2014)	2.825	0.707	0.499	1.440	4.210	3.998	0.000
	Park et al. (2011)	19.037	3.043	9.260	13.073	25.002	6.256	0.000
	Sunoo and Hwang (2004)	0.790	0.537	0.289	-0.263	1.843	1.470	0.142
	Sunoo et al. (2005)	3.771	0.786	0.617	2.231	5.311	4.800	0.000
	Jung et al. (2004)	1.511	0.507	0.257	0.518	2.505	2.981	0.003
	Kim et al. (2009)	15.064	1.980	3.921	11.183	18.945	7.608	0.000
Random		5.447	1.234	1.523	3.028	7.866	4.414	0.000

Heterogeneity: Q-value=119.043(p=.000), I^2^=94.120

**Table 4.** Effects of altitude/hypoxic training vs. sea-level training on Hct (%).

Model	Study name	Std diff in means	standard error	variance	Lower limit	Upper limit	Z-value	p-value
	Sunoo et al. (2007)	4.834	0.864	0.747	3.140	6.528	5.592	0.000
	Shin and Cho (2003)	7.217	1.370	1.878	4.531	9.903	5.267	0.000
	Yun and Lee (2014)	3.726	0.827	0.684	2.105	5.347	4.506	0.000
	Park et al. (2011)	2.285	0.575	0.331	1.158	3.412	3.974	0.000
	Sunoo and Hwang (2004)	0.222	0.519	0.270	-0.795	1.240	0.428	0.669
	Sunoo et al. (2005)	1.102	0.506	0.256	0.111	2.094	2.178	0.029
	Jung et al. (2004)	0.899	0.469	0.220	-0.021	1.819	1.916	0.055
	Kim et al. (2009)	56.258	7.272	52.888	42.004	70.511	7.736	0.000
Random		3.639	0.996	0.992	1.687	5.591	3.654	0.000

Heterogeneity: Q-value=104.973(p=.000), I^2^=93.332

**Table 5.** Effects of altitude/hypoxic training vs. sea-level training on EPO (mU/mL).

Model	Study name	Std diff in means	standard error	variance	Lower limit	Upper limit	Z-value	p-value
	Sunoo et al. (2007)	0.369	0.441	0.194	-0.495	1.233	0.837	0.402
	Yun and Lee (2014)	1.210	0.544	0.296	0.144	2.275	2.224	0.026
	Park et al. (2011)	0.958	0.472	0.223	0.032	1.883	2.029	0.042
	Sunoo and Hwang (2004)	0.384	0.522	0.273	-0.639	1.408	0.736	0.462
	Sunoo et al. (2005)	0.752	0.488	0.238	-0.204	1.708	1.542	0.123
Fixed		0.711	0.219	0.048	0.282	1.140	3.251	0.001

Heterogeneity: Q-value=2.115(p=.715), I^2^=0.000

### The effect of altitude/hypoxic training on the aerobic exercise capacity

Seven studies were selected for the effect of altitude/hypoxic training on VO_2_max. Heterogeneity was identified in VO_2_max (Q-value = 56.328, p = .000, I^2^ = 89.348) and effect size calculated by random effect model. Elite athletes in the altitude/hypoxic training group improved their VO_2_max by 1.637 ml/kg/min (95% CI: 0.599 - 1.400, p=.000) more than the sea-level training group (Table 6).

**Table 6.** Effects of altitude/hypoxic training vs. sea-level training on VO_2_max (ml/kg/min).

Model	Study name	Std diff in means	standard error	variance	Lower limit	Upper limit	Z-value	p-value
	Sunoo et al. (2007)	0.171	0.438	0.192	-0.687	1.029	0.390	0.697
	Shin and Cho (2003)	0.313	0.503	0.253	-0.673	1.299	0.622	0.534
	Yun and Lee (2014)	1.425	0.560	0.313	0.328	2.523	2.546	0.011
	Sunoo and Hwang (2004)	0.585	0.528	0.279	-0.451	1.620	1.106	0.269
	Sunoo et al. (2005)	0.200	0.473	0.223	-0.726	1.126	0.423	0.672
	Jung et al. (2004)	2.832	0.633	0.401	1.592	4.073	4.475	0.000
	Kim et al. (2009)	7.232	1.005	1.011	5.262	9.202	7.194	0.000
Random		1.637	0.641	0.411	0.381	2.894	2.554	0.011

Heterogeneity: Q-value=56.328(p=.000), I^2^=89.348

## DISCUSSION

A number of studies have been conducted on the effect of altitude/hypoxic training on oxygen delivery capacity of the blood and aerobic exercise capacity. These studies reported inconsistent results (positive and negative results) due to difference in physiological characteristics and training conditions (method, intensity, frequency, duration, and time of training)^26^. Altitude/hypoxic environments training has positive effects on VO_2_max, oxygen consumption (VO_2_), maximum ventilation, Hb, EPO, area of capillary blood vessel, 2,3-diphosphoglycerate (DPG), density of mitochondria, storage of glycogen, muscle buffer capacity, lactate threshold, strength and power, psychological limitation, hypoxic inducible factor (HIF)-1, vascular endothelial growth factor (VEGF), and glycolysis enzyme^2, 14, 22, 29, 35, 42, 47, 49^. Together with positive effects, altitude/hypoxic environments training leads to negative effects on blood viscosity, muscle blood flow, cardiac output, HRmax, protein synthesis, and Na+-K+-ATPase activity, and decreases training quality and quantity^5, 21, 28, 31^. These controversial effects are dependent on sports event, performance level, support of nutrition and medical, fatigue level, training type, physiological state, psychological state of subject, and lead to inconsistent of study results.

Due to these positive or negative effects of altitude/ hypoxic environments training, athletes, coaches, trainers, and researchers have continued argument about effect in oxygen delivery capacity of the blood and aerobic exercise capacity. In this study, we accordingly conducted meta-analysis on 8 research studies with elite athletes in Korea to verify practical applicability of altitude/hypoxic environments training, and propose the direction of training system development for athletic performance. Our results indicated that altitude/hypoxic environments training is more efficient than sea-level training in terms of oxygen delivery capacity of the blood (RBC, Hb, Hct, EPO) and aerobic exercise capacity (VO_2_max). Although, training type, exercise intensity, frequency, and duration were different in 8 research studies for meta-analysis, the result shows that training of more than 3 weeks, 3 times a week, and 1 hour can improve oxygen delivery capacity of the blood and aerobic exercise capacity. Also, the result is in agreement with previous meta-analysis studies that reported altitude/hypoxic environment training enhances aerobic exercise capacity and performance through increase of RBC mass, Hb mass and VO_2_max^3^ and exposure of altitude/ hypoxic environment increases Hb mass in 2 weeks and improves aerobic exercise capacity and performance^11^. An increase in investment by administration department for various types of altitude/hypoxic environments training facility (e.g. hypobaric/hypoxic lodging, hotel, mask, training truck, training center etc.) is needed for improved aerobic exercise capacity and performance among elite athletes in Korea. In addition, members of the athletic community including athletes, managers, and coaches should change the perception of altitude/hypoxic training and apply to elite athletes. Currently, U.S.A. has established Colorado Altitude Training (CAT) and possesses hypoxic tent, hypoxic hotel, hypoxic room, hypoxic swimming pool, hypobaric and hypoxic training center. Through altitude/ hypoxic training in the facilities, long distance speed skating team in 2002 Salt Lake City Winter Olympic & 2006 Torino Winter Olympic and marathon team in 2004 Athens Olympic reported unprecedented success^45^. Japan also has founded Japan Institute for Sports Science (JISS) in 2002 and possesses hypoxic room, hypoxic swimming pool, and hypobaric/hypoxic training center. In Japan, various events’ athletes have conducted hypobaric/hypoxic training and maintained high athletic performance since the 2004 Athens Olympics^26^. These facts prove that investment in altitude/hypoxic training facility and efforts to apply altitude/ hypoxic training are required acutely for increase of elite athletes and national competitiveness in sports.

Detail examination of previous studies applied in a meta-analysis reveals that elite athletes’ events are different (high school soccer players, high school runners, national team level pin swimmers, college runners, college basketball players) and applied altitude/hypoxic environments training type are also distinguished into 2 studies of LHTH^7, 38^, 1 study of LHTL^26^, and 5 studies of LLTH^15, 36, 39, 40, 48^. Therefore, in results of meta-analysis, variables except EPO showed heterogeneity of effect sizes. The results indicated that altitude/hypoxic environment training is more efficient than sea-level training in terms of oxygen delivery capacity of the blood and aerobic exercise capacity, but they cannot explain altitude/hypoxic environments training type, exercise intensity, frequency, and duration for increase of athletic performance. Therefore, meta-analysis should be conducted for various dependent variables, aerobic exercise capacity, and athletic performance after classification according to altitude/hypoxic environment training type and athletes’ event in previous Korean and other studies. These efforts would identify the most effective altitude/hypoxic environment training for increased oxygen delivery capacity of the blood, aerobic exercise capacity, and athletic performance.^2^

## CONCLUSIONS

This study was designed as a meta-analysis of randomized controlled trials comparing effectiveness of altitude/ hypoxic training versus sea-level training on oxygen delivery capacity of the blood and aerobic exercise capacity of elite athletes in Korea. It comprised a five step process of the PRISMA flowchart after setting the selection criteria for the study based on PICOS introduced in the Cochrane guideline. Homogeneity was identified in EPO but heterogeneity was identified in RBC, Hb, Hct, and VO_2_max due to difference in the pattern of sporting event and altitude/ hypoxic training type between each study. RBC, Hb, Hct, EPO, and VO_2_max were significantly increased following altitude/hypoxic training, as compared with sea-level training. For elite athletes in Korea, altitude/hypoxic training appears more effective than sea-level training for improvement of oxygen delivery capacity of the blood and aerobic exercise capacity. Therefore, increased investment in the various altitude/hypoxic training facilities (hypobaric and hypoxic room, hotel, mask, training truck, training center), change of awareness and application of altitude/hypoxic training are needed for improvement of athletic performance in elite athletes.
